# Artificial intelligence for climate–health early warning systems in the Horn of Africa: opportunities, challenges, and a roadmap for action

**DOI:** 10.1186/s12992-026-01233-9

**Published:** 2026-08-08

**Authors:** Ahmed Abdiaziz Alasow, Yusuf Hared Abdi, Abdifatah Ahmed Hersi, Maxwell Boakye

**Affiliations:** 1 Department of Environmental Science and Technology, Faculty of Forestry and Environment, University Putra Malaysia, Serdang, Selangor 43400 Malaysia; 2 Department of Public Health, Faculty of Medicine and Health Science, Hormuud University, Mogadishu, Somalia; 3 Faculty of Economics and Management Sciences, Hormuud University, Mogadishu, Somalia; 4 Department of Environment and Public Health, School of Natural and Environmental Sciences, University of Environment and Sustainable Development, Akropong, Ghana

**Keywords:** Artificial intelligence, Climate–health early warning systems, Horn of Africa, Digital surveillance, Health systems resilience

## Abstract

Climate extremes, conflict, and population displacement converge in the Horn of Africa to accelerate outbreaks of climate-sensitive infectious diseases, whereas existing health surveillance systems remain fragmented and largely reactive. This Perspective examines the potential of artificial intelligence (AI) to strengthen climate–health early warning by integrating satellite earth observations, routine disease surveillance, and mobility-based vulnerability indicators into anticipatory decision support systems. Drawing on global experience and region-specific constraints, we identified critical barriers to implementation, including data fragmentation, infrastructure gaps, workforce shortages, governance silos, and unresolved ethical risks. We propose a five-layer conceptual framework for an AI-enabled Climate–Health Early Warning System (CHEWS) tailored to fragile and conflict-affected settings, alongside a phased regional policy roadmap anchored within the Intergovernmental Authority on Development (IGAD). Emphasizing data sovereignty, participatory governance, and privacy-by-design, this study positions AI-CHEWS as a feasible pathway for shifting the region from reactive outbreak responses to anticipatory public health actions that enhance climate resilience and equity.

**Clinical Trial Number:** The authors declare that they have no competing interests.

## Introduction: climate–health urgency

The Horn of Africa—comprising Djibouti, Eritrea, Ethiopia, Kenya, Somalia, South Sudan, Sudan, and Uganda—is currently navigating a ‘poly-crisis’ where the converging forces of climate change, conflict, and fragile health systems are accelerating disease emergence at a pace that outstrips conventional surveillance capacities [1]. Between 2020 and 2024, the region faced the most severe drought in 40 years, followed immediately by catastrophic El Niño flooding, creating a volatile incubator for climate-sensitive infectious diseases (CSIDs) [2, 3]. This hydrological whiplash has driven concurrent outbreaks of cholera in Somalia and Ethiopia, dengue fever in Sudan, and malaria upsurge in the highlands of Kenya [4, 5]. In this context, the traditional “passive” surveillance approach, which relies on retrospective case counting at health facilities, is structurally unsuccessful [6]. By the time a cholera cluster is confirmed in a remote displacement camp in Baidoa or a malaria spike is registered in Jonglei, the window for effective containment is often closed [7]. Crucially, this ‘poly-crisis’ is not merely a localized convergence of misfortunes but a manifestation of structural global inequities. The Horn of Africa contributes less than 4% of global greenhouse gas emissions yet bears a disproportionate share of the catastrophic health consequences—a clear instance of climate injustice driven by globalized industrial activities [8]. Furthermore, the region’s instability is often exacerbated by global geopolitical competition and resource extraction dynamics, which prolong conflict and displacement, thereby deepening vulnerability to climate shocks. Consequently, the failure of health systems here is not solely a domestic deficit but partly a legacy of global economic policies, including structural adjustment programs that have historically eroded public sector capacity and deepened reliance on volatile external aid [9].

A critical limitation of current systems exacerbated by fragmented funding streams and donor-driven priorities is their inability to integrate multi-hazard signals before an outbreak occurs [1, 10]. While meteorological agencies such as the IGAD Climate Prediction and Applications Center (ICPAC) possess sophisticated satellite data on rainfall anomalies and Ministries of Health maintain disease records, these data streams remain siloed [1]. There is no automated mechanism for fusing real-time environmental precursors (soil moisture and temperature suitability) with vulnerability indicators (acute malnutrition and population displacement) to generate predictive alerts [11]. Consequently, health responses remain reactive and deployed only after the morbidity and mortality thresholds have been breached [6].

Artificial Intelligence (AI) offers a mature technological bridge to this gap. The rapid evolution of machine learning (ML) now allows for the fusion of heterogeneous datasets combining satellite earth observations (EO), anonymized mobility patterns from cellular networks, and clinical data to forecast outbreaks weeks or months in advance [12, 13]. However, despite the global proliferation of AI tools, the Horn of Africa lacks a coordinated, region-specific AI-enabled climate-health early warning system (CHEWS) [1, 14]. This article argues that developing such a system is not merely a technical upgrade, but a humanitarian imperative. We outline the opportunities for an AI-driven CHEWS within the IGAD framework, analyze the specific barriers in fragile and conflict-affected settings, and propose a roadmap to operationalize this technology for anticipatory action.

## Global evolution of ai for climate–health early warning

The application of AI to climate-sensitive disease forecasting has transitioned from academic experimentation to operational reality in several regions, thereby offering critical lessons for the Horn of Africa [15, 16]. Globally, the shift has been from simple regression models to complex machine learning architectures capable of capturing nonlinear relationships between climate variables and disease transmission [15, 17].

Artificial Intelligence has revolutionized dengue forecasting in Southeast Asia and Latin America [16]. Brazil’s InfoDengue system—a web-based early warning platform—is a premier example, utilizing a hybrid model that integrates meteorological data, social media analysis, and clinical notifications to produce weekly risk maps [12]. Similarly, in Singapore, forecasting models using the Least Absolute Shrinkage and Selection Operator (LASSO) and Support Vector Machines (SVM) have successfully predicted dengue trends up to three months in advance, allowing vector control teams to preemptively target breeding sites [18]. For malaria, the focus has shifted toward “last-mile” prediction in resource-limited settings [7]. The Malaria Anticipation Project in South Sudan, piloted by Médecins Sans Frontières, demonstrated the viability of using focused environmental data to predict caseloads in conflict zones, whereas the iDEWS (integrated Disease Early Warning System) project in Ethiopia has explored integrating precipitation lags into district-level planning [7, 10].

Technically, the field coalesces around ensemble methods [17, 19]. Random Forest and XGBoost algorithms have become industry standards for classification tasks owing to their robustness against noisy data, which is a common feature in low-resource health systems [7, 19]. Concurrently, Long Short-Term Memory (LSTM) networks are increasingly deployed for time-series forecasting, excelling in retaining long-term climatic patterns, such as the El Niño Southern Oscillation (ENSO) [20, 21]. Furthermore, the emergence of Geospatial Artificial Intelligence (GeoAI) has enabled granular risk mapping at the village level, which is crucial for identifying “hotspots” rather than generalized district-level alerts [19, 22].

The most pertinent lesson for the Horn of Africa from these global experiences is the necessity of integrating non-climatic variables. European heat-mortality systems have shown that temperature data alone are insufficient without demographic vulnerability layers (e.g., age and housing quality) [23]. Similarly, cholera prediction models in Yemen and Bangladesh have highlighted that hydroclimatic data must be coupled with water, sanitation, and hygiene (WASH) infrastructure data to be actionable [24]. For the Horn of Africa, this implies that any successful AI model must ingest data on human displacement and conflict intensity, as these factors often override the pure climatic drivers in precipitating outbreaks [25].

## Gaps, challenges & risks in the Horn of Africa

Implementing AI-driven CHEWS in the Horn of Africa faces systemic barriers that are distinct from stable, high-income contexts [1]. The primary challenge is the “data division.” While satellite data are ubiquitous, ground-truth epidemiological data are often fragmented, paper-based, or non-existent in areas controlled by non-state-armed groups [1, 6]. In Sudan, conflict has severed reporting lines, creating “surveillance black holes, where AI models effectively fly blind [25]. Furthermore, the historical health data essential for training algorithms often lack the temporal consistency required to minimize bias, leading to models that may overfit recent anomalies rather than long-term trends [6].

Infrastructure remains a challenging constraint. High-latency Internet connectivity in rural Ethiopia and South Sudan impedes the real-time upload of facility data to central cloud repositories [26]. This “digital cliff” means that sophisticated central models may generate alerts that cannot reach the peripheral health workers who need them most [14]. Additionally, the region suffers from a critical shortage of ‘climate epidemiologists’ professionals cross-trained in data science, public health, and meteorology [6]. Reliance on external consultants often leads to “black box” models that local Ministries of Health cannot maintain or audit once donor funding cycles conclude.

Fragile and conflict-affected settings present unique implementation challenges that differentiate the Horn of Africa from stable, high-income contexts where most AI-CHEWS literature originates. In Somalia and South Sudan, active conflict has fragmented governance, with non-state armed groups controlling territories where government health systems have no presence. These “surveillance black holes” mean training data for AI models are systematically biased toward government-controlled urban centers, while outbreak risk is highest in inaccessible rural and displaced populations. In Sudan’s ongoing conflict, health facilities have been deliberately targeted, destroying both paper records and nascent digital infrastructure. Libya, though geographically in North Africa, faces similar challenges: political fragmentation between rival governments undermines unified disease surveillance, and cross-border population movements link Libyan instability to Horn of Africa epidemiology (e.g., migrants transiting to/from Eritrea and Ethiopia). The political economy of these conflicts further complicates AI-CHEWS: governments may resist transparent outbreak reporting if data could be weaponized politically (e.g., opposition groups citing health failures) or if international attention triggers unwanted humanitarian access demands in contested territories. Therefore, AI-CHEWS deployment in fragile states requires conflict-sensitive design: federated architectures where data never leave jurisdictional boundaries, neutral regional hosting (IGAD/Africa CDC rather than bilateral donors), and alert systems that function even when national governments are non-functional—routing directly to WHO/NGO humanitarian clusters [25, 27].

Governance fragmentation further complicates the integration. Climate data are based on meteorological agencies (such as ICPAC or national Met services), while health data reside with the Ministry of Health [1]. These entities rarely share data protocols or interoperable application programming interface (API) standards. In the absence of a binding regional data-sharing framework, crucial environmental signals are not operationalized for health [27]. Finally, the ethical dimension of conflict zones is acute. AI models relying on tracking population movement via call detail records (CDRs) pose significant privacy risks for displaced populations fleeing persecution [28]. There is a tangible risk that surveillance infrastructure built for health can be used for security or political control [29]. Beyond privacy and bias, a more fundamental challenge is the risk of ‘digital colonialism.’ Currently, the global AI landscape is dominated by technology firms in the Global North, creating a dependency where African nations provide the raw data while the algorithmic value and intellectual property are captured abroad. In the absence of robust data sovereignty frameworks, there is a danger that CHEWS could function as extractive mechanisms, where health and mobility data from vulnerable populations are processed by proprietary ‘black box’ models over which local health ministries lack control. This power asymmetry threatens to lock the Horn of Africa into a cycle of technological dependence, limiting the region’s ability to adapt systems to local realities and prioritize indigenous public health agendas over external commercial or security interests. These structural, infrastructural, governance, ethical, and financial barriers are summarized in Table 1.

**Table 1:** AI CHEWS barriers

Domain	Key Challenges	Mitigation Strategies
Data	Fragmented paper-based reporting Integrated Disease Surveillance (IDSR); lack of interoperability between climate & health datasets; “black holes” in conflict zones; missing values > 40% in South Sudan/Somalia; inconsistent reporting delays (2–4 weeks) [6, 25].	• Data harmonization protocols through IGAD framework • Implement District Health Information Software 2 (DHIS2) mobile app for real-time reporting • Multiple imputation techniques for missing data • Satellite-based proxy indicators for conflict zones • Incentivize facility reporting through data quality audits
Infrastructure	Low internet penetration in rural/pastoralist areas (< 30% connectivity); absence of sovereign cloud infrastructure; reliance on unstable power grids; high-latency networks impede real-time data upload	• SMS-based alert dissemination (low-bandwidth) • Hybrid on-premise/cloud architecture • Solar-powered data collection devices • Edge computing for local processing • Regional IGAD data centers for sovereignty
Human Resources	Severe shortage of local data scientists and climate epidemiologists (< 50 across region); “brain drain” of technical talent to private sector/abroad; limited cross-training in meteorology + epidemiology; low retention in public sector [6, 26].	• Dedicated Health Informatician civil service cadre with competitive salaries • Regional training hubs at universities • South-South technical exchanges (Kenya-Somalia) • Research time allocation for retention • Open-source tools requiring less coding expertise
Governance	Siloed operations between Ministries of Health and Meteorological agencies; lack of cross-border data sharing protocols; absence of binding regional agreements; competing bureaucratic priorities; unclear decision-authority for alert triggers [1].	• Joint ministerial steering committees (Health-Meteorology-ICT) • IGAD data sharing treaty with legal force • Standardized APIs between climate and health systems • Clear standard operating procedures linking alerts to response • Regional coordinating body (IGAD/Africa CDC)
Cybersecurity	Vulnerability to ransomware attacks on health databases; risk of system manipulation during conflicts; limited cybersecurity expertise in health ministries; potential for adversarial attacks on AI models (poisoning training data)	• End-to-end encryption for data transmission • Multi-factor authentication for system access • Regular security audits by regional/international experts • Offline backup systems (air-gapped) • Cyber incident response protocols • Training health IT staff in security best practices
Data Protection & Privacy	Risks of deanonymization in displacement tracking; potential dual-use of surveillance data by security forces; inadequate data protection legislation in some countries; geospatial data revealing vulnerable settlement locations; CDR data exposing individual movements [28, 29].	• Privacy-by-design architecture (data minimization) • Aggregate-only reporting (no individual-level data shared) • Differential privacy techniques • Coarsen geospatial resolution (district, not village) • Legal firewalls preventing security access • African Union Cyber Security Convention compliance • Independent ethics review boards
Ethics	Algorithmic bias against marginalized groups (refugees, pastoralists); historical underreporting creates prediction gaps; power asymmetries (Global North algorithms, African data); digital colonialism risks—data extracted, value captured abroad; risk of alert fatigue and false positives eroding trust [29].	• Bias audits using representative validation datasets • Participatory model development with affected communities • Local data science teams (not offshore consultants) • Federated learning (data sovereignty preserved) • Transparent model documentation • Contextual threshold calibration by frontline workers • Sentinel surveillance in underserved areas
Regulatory & Legal	Absence of regional AI governance frameworks; unclear liability for incorrect predictions; lack of certification standards for AI health tools; incompatible national data protection laws; legal barriers to cross-border data flows; no licensing framework for AI diagnostic tools [27].	• IGAD regional AI-CHEWS standards and certification • Legal liability framework (good-faith exemptions for public health use) • Harmonize data protection laws (AU-level advocacy) • Mutual recognition agreements for cross-border data • National AI ethics guidelines adapted to health context • WHO AFRO regional technical standards
Financing	Project-based, short-term donor funding prevents long-term infrastructure capability; lack of domestic budget lines for digital health maintenance (< 2% surveillance budgets); competing priorities (immediate humanitarian response vs. upstream prevention); climate finance underutilized for health adaptation; uncertain sustainability beyond pilot phases [6, 27].	• IGAD Climate-Health Trust Fund (pooled, multi-year) • Domestic budget mandates (≥ 5% health budget to surveillance) • Anticipatory financing instruments (parametric insurance) • Climate adaptation finance (Green Climate Fund) • Private sector co-investment (telecoms, cloud providers) • Demonstrate cost-benefit ($1 prevention saves $10 response) • Phased rollout (prove value before full investment)
Rural/Remote Settings	Lack of existing AI and robust EWS in rural and remote settings where most needed; last-mile connectivity challenges; limited electricity for device charging; health worker digital literacy gaps; pastoralist mobility complicates surveillance; geographic inaccessibility during floods [14, 26].	• Community-based participatory surveillance (CHW SMS reporting) • USSD codes (no smartphone required) • Solar charging stations at health posts • Simplified interfaces with visual icons • Train-the-trainer cascade to CHW level • Predictive pre-positioning to remote areas before access cutoff • Leverage mobile operators’ coverage expansion

## Conceptual framework for AI-enabled CHEWS

**Fig. 1:**
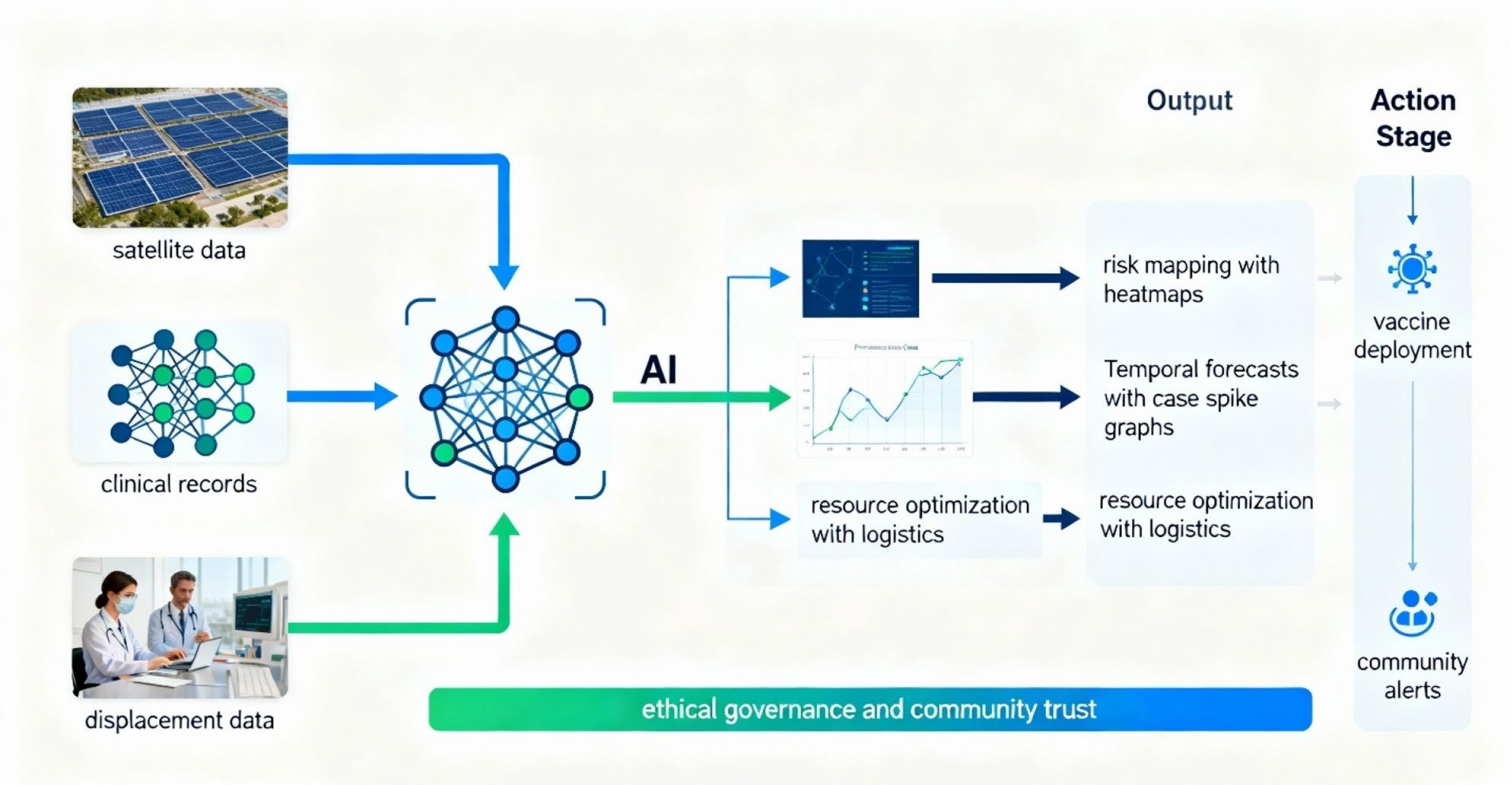
Conceptual flow diagram

To overcome these barriers, we propose a modular five-layer CHEWS architecture, designed specifically for the resource constraints of the region. This framework moves beyond simple correlation to a dynamic end-to-end early warning pipeline. The proposed multilayer CHEWS architecture is illustrated in Fig. 1.

The foundation Climate Hazard Detection Layer operates independently of the health system, ingesting open-source Earth Observation (EO) data [30]. This includes precipitation estimates (CHIRPS), land surface temperature (MODIS/Landsat), and vegetation indices (NDVI) processed by regional hubs, such as ICPAC [1, 4]. This layer provides the “environmental risk envelope”. Above this sits the exposure and vulnerability layers, which contextualize the hazard. In the Horn of Africa, this layer is critical and must integrate dynamic data: displacement tracking matrices (DTM) from the IOM, nutritional surveillance data (IPC acute malnutrition rates), and WASH coverage maps [31]. AI techniques were used to interpolate missing data points in conflict-affected areas [15].

The core intelligence resides in the AI Analytics Layer. Ensemble machine learning models (e.g., Random Forest or XGBoost) fuse the hazard and vulnerability streams to classify risk probabilities [7]. For example, a model might identify that “above-average rainfall” (Hazard) combined with “high displacement” (Vulnerability) and “low cholera vaccination coverage” creates a 90% probability of an outbreak. This output feeds the decision support layer, which translates the probabilities into operational alerts [18]. Crucially, this layer is not just a dashboard but also a logistics trigger automated alert sent to district health officers proposing specific anticipatory actions, such as pre-positioning oral rehydration salts or initiating vector control [7]. Finally, the Governance and Ethics Layer wraps the entire system, ensuring that algorithms are audited for bias, and data privacy standards such as the African Union Convention on Cyber Security are enforced [28].

### Feature engineering for climate-health linkages

Raw meteorological variables (rainfall, temperature) rarely predict disease incidence directly; instead, derived features capture biological mechanisms. For malaria, lagged precipitation (typically 4–8 weeks prior) combined with temperature suitability indices (e.g., Detinova’s sporogonic cycle model) provide stronger predictive signals than raw values. For cholera, cumulative rainfall over 7-day windows correlates with flooding events that contaminate water sources. Feature engineering must also encode vulnerability: distance to nearest health facility, IPC malnutrition phase classification, and IOM displacement tracking matrix data become critical covariates that contextualize climatic risk [4, 10].

### Model selection: ensemble vs. deep learning

Given the limited historical data availability (< 5 years of consistent surveillance in many districts), ensemble tree-based methods (Random Forest, XGBoost, LightGBM) typically outperform deep learning approaches in the Horn of Africa context [7, 19]. These models handle missing data better, require less training data, and provide interpretability through feature importance rankings—crucial for building trust with Ministries of Health [7, 19]. Deep learning approaches (LSTM networks, Transformer models) may be viable for countries with longer time series (e.g., Kenya malaria data dating to 2000) but risk overfitting in data-scarce Somalia or South Sudan. A pragmatic approach is to deploy ensemble methods for operational forecasting while investing in long-term data infrastructure that will eventually enable more sophisticated architectures [7, 19].

### Model training challenges: data fragmentation and class imbalance

Practical training faces region-specific challenges. First, jurisdictional data fragmentation where district-level case data cannot be legally shared across borders prevents training unified regional models, necessitating federated learning approaches where algorithms train on decentralized data without raw data transfer. Second, extreme class imbalance (outbreak weeks represent < 5% of total observations) causes models to default to “no outbreak” predictions. Techniques such as Synthetic Minority Oversampling (SMOTE) or cost-sensitive learning that penalizes false negatives more heavily are essential [15, 27].

### Validation in non-stationary environments

Standard k-fold cross-validation is insufficient in climatic contexts where El Niño cycles introduce non-stationarity. Time-series cross-validation with expanding windows—where models trained on 2015–2020 data are tested on 2021–2022—better simulates operational reality. Furthermore, spatial cross-validation (training on Kenya/Ethiopia, testing on Somalia) assesses whether models generalize across borders, a critical requirement for regional IGAD deployment [5, 11, 32].

### Technical infrastructure requirements

Model training requires computational resources currently lacking in most Horn of Africa institutions. A single Random Forest model with 500 trees, trained on 50,000 observations with 100 features, requires ~ 16GB RAM and 2–4 h on a modern CPU. Cloud-based solutions (AWS SageMaker, Google Vertex AI) provide scalability but raise data sovereignty concerns. A hybrid approach—preprocessing and validation on local university servers, with model training burst-computing on encrypted cloud instances—balances sovereignty and capability. Critical is establishing regional data centers (potentially hosted by IGAD or Africa CDC) that provide computing infrastructure without data exfiltration to Global North servers [12, 29, 30].

### Open-source tooling and capacity building

To prevent vendor lock-in, all model development should utilize open-source frameworks: Python’s scikit-learn for ensemble methods, TensorFlow/PyTorch for deep learning, and QGIS for geospatial feature extraction [19, 30]. Training curricula for Ministries of Health staff should prioritize hands-on model auditing rather than algorithm development from scratch, ensuring sustainability after external consultants depart [31, 33].

## Opportunities for AI implementation

Despite these challenges, there are immediate high-impact entry points for AI deployment in the region. The most feasible opportunity lies in the integration of remote sensing for vector-borne diseases [4]. Malaria and the Rift Valley Fever (RVF) have strong environmental signatures. AI models can be trained on historical NDVI (vegetation) and rainfall data to predict breeding site suitability with high accuracy weeks before the vectors emerge [4]. This requires no new ground infrastructure and only the computational capacity to process existing satellite feeds and disseminate SMS alerts to district officers [30].

Digital Surveillance offers another “leapfrog” opportunity. The widespread adoption of mobile phones allows for participatory surveillance systems in which community health workers (CHWs) report syndromic data (e.g., “number of children with watery diarrhea”) via USSD codes [25, 33]. AI natural language processing (NLP) can be applied to aggregate unstructured reports, identifying anomalous clusters faster than formal paper trails [34]. In refugee camps, analyzing anonymized mobile network operator data can model population flux, allowing aid agencies to predict where susceptible hosts are moving into high transmission zones [13, 35].

Preparedness Optimization represents the highest return on investment. Instead of predicting the exact number of cases, AI can optimize the location of the response assets [7]. “Hotspot forecasting” allows Ministries of Health to pre-position vaccine stockpiles and cholera kits in regional hubs likely to be cut off by floods [36]. This shift from “responding to outbreaks” to “anticipatory humanitarian action” aligns with global financing shifts, such as the release of funds based on pre-agreed forecast triggers [7]. Regional Scaling via IGAD provides political vehicles for these tools [1]. By pooling data across borders, a regional AI model can track cross-border transmission corridors essential for pastoralist communities moving between Ethiopia, Kenya, and Somalia, creating a collective security shield that no single nation can build alone [27].

### Integration with existing IDSR and EWS infrastructure

AI-CHEWS cannot function as a parallel system; it must augment existing Integrated Disease Surveillance and Response (IDSR) frameworks mandated by the World Health Organization and operational across the Horn of Africa since 1998 [6]. Deployment strategies must prioritize interoperability, minimal disruption to current workflows, and phased scaling [26, 37].

### DHIS2 integration: leveraging existing digital infrastructure

The District Health Information Software 2 (DHIS2) platform currently used by all Horn of Africa countries for health data aggregation provides the most viable integration point [37]. DHIS2’s modular architecture allows custom applications to be embedded without replacing core functionality [16]. The proposed approach involves developing a DHIS2 “CHEWS module” that:

Automatically ingests weekly case reports already entered by district health officers, ensuring no additional data entry burden [10]. Pulls climate data from ICPAC APIs in the background using scheduled scripts [30]. Runs AI risk algorithms on a backend server, then pushes alert flags back into DHIS2 dashboards [11].

Displays color-coded risk maps (green/yellow/red) within the familiar DHIS2 interface health workers already use daily [15]. This approach requires no retraining of frontline staff on new software and preserves the IDSR reporting chain of command [6]. Technically, it requires deploying Python-based prediction scripts on a server (on-premise or cloud) that communicates with DHIS2 via its REST API [30].

### SMS alert integration for low-connectivity settings

In remote pastoralist regions lacking reliable internet, such as Turkana, Afar, or rural Somalia, AI-generated alerts must reach health workers via SMS rather than dashboards [14]. Integration with existing SMS gateways like RapidPro or FrontlineSMS, which are already used for vaccine campaign notifications, allows alerts to piggyback on trusted communication channels [25, 33]. The system architecture involves:

A central AI model that generates district-level risk scores on a weekly basis [18].

An automated script that queries the score and triggers an SMS if a specific threshold is exceeded, such as a 70% cholera risk [38].

SMS content providing actionable, pre-approved messages, such as instructions to pre-position oral rehydration salts (ORS) and activate rapid response teams [7].

Two-way SMS capabilities that allow health workers to report “surveillance zero” or identify early case clusters [25].

Integration with Meteorological Early Warning Systems.

ICPAC already issues seasonal climate forecasts to governments, but these often lack health-specific translation [1]. AI-CHEWS creates the missing bridge through the following:

Automated ingestion where the AI system subscribes to ICPAC’s Climate Outlook Forum bulletins via API [1].

A translation layer using machine learning models to convert generalized climate forecasts into disease-specific risks, such as the probability of a malaria surge following above-normal rainfall [4, 5].

Joint bulletins where monthly co-branded ICPAC-Ministry of Health “Climate-Health Bulletins” are distributed to district officers [24].

This leverages ICPAC’s established credibility while adding epidemiological specificity that is currently absent [1].

### Enhancing WHO EWARN in conflict settings

In active conflict zones like Sudan and Somalia, the WHO Early Warning, Alert and Response Network (EWARN) uses simplified syndromic reporting rather than full IDSR [25]. AI integration here involves:

Anomaly detection algorithms that flag unusual syndromic surges automatically, reducing the reliance on manual threshold interpretation by overstretched staff [38].

Predictive pre-alerts sent to humanitarian clusters 1–2 weeks before forecasted high-risk periods to enable the pre-positioning of emergency supplies [7].

Mobile data integration where anonymized call detail records (CDRs) can track population displacement into high-risk zones to trigger targeted surveillance [35].

### Capacity building for operationalization

Technical integration is insufficient without workforce capacity [31]. The deployment strategy includes:

Embedded Data Analysts: Placing one AI-trained epidemiologist in each national Ministry of Health to serve as an AI-CHEWS focal point responsible for model monitoring and alert translation [28].

District Training Cascade: Three-day workshops for district health officers on interpreting AI risk maps and triggering response protocols [33].

Learning Networks: Quarterly virtual “Community of Practice” meetings where health officers share lessons on which alert thresholds work best operationally [37].

Phased Rollout Strategy.

Rather than region-wide simultaneous deployment, a phased approach should be adopted to mitigate the risk of failure [11].

Phase 1 (Year 1): Pilot integration in 3–5 border districts with robust IDSR systems, such as Garissa-Kenya or Baidoa-Somalia [10].

Phase 2 (Year 2): Based on pilot lessons, expand the system to 10–15 high-burden districts across all IGAD countries [18].

Phase 3 (Year 3+): National-scale rollout with automated data pipelines fully established [26].

Each phase includes rigorous evaluation to determine if AI alerts reduced response times and to identify operational barriers.

## Ethics, equity & governance

The deployment of AI in humanitarian contexts carries profound ethical risks that must be preemptively managed to prevent “data colonialism” which involves the extraction of data from vulnerable African populations to train algorithms owned by Global North institutions without local benefit [28]. There is a specific risk of algorithmic bias against displaced communities; if historical data from refugee camps are poor (due to lack of access), models may systematically underpredict outbreaks in these populations, leading to resource allocation that favors stable, better-monitored urban centers [28].

Thus, political misuse is a significant threat. The volatile security landscape, precise data on population movements and resource vulnerabilities are strategically valuable [29]. There must be firewalls ensuring that health early warning data cannot be accessed by military or intelligence actors for targeted purposes [38]. Furthermore, the exclusion of national researchers from the model development process created a dependency trap. Systems built by fly-in consultants often fail once the contract ends because the “intellectual ownership” remains offshore.

Therefore, governance principles must prioritize Data Sovereignty. Member states should retain ownership of their health data, with AI models trained via federated learning approaches that allow algorithms to learn from decentralized data without moving raw sensitive records across borders [37]. Participatory Modeling is equally vital; pastoralist communities and frontline health workers must be involved in defining the “alert thresholds” to ensure that the system generates warnings that are culturally and operationally relevant [32]. Finally, Privacy-by-Design standards must be strictly enforced, particularly regarding geospatial resolution, to ensure that mapped “hotspots” cannot be used to pinpoint vulnerable settlements in conflict zones [38].

### Capacity building and technology transfer framework

Sustainable AI-CHEWS deployment hinges on building indigenous technical capacity within the Horn of Africa, rather than perpetuating dependence on external consultants [29]. The region currently faces severe shortages, with fewer than 50 professionals trained in both public health and data science across all eight IGAD member states, and no university currently offers a dedicated “Climate Health Informatics” program [1, 26]. Addressing this requires a comprehensive, multi-layered capacity development strategy that aligns with continental digital health priorities [37]. Addressing these gaps is critical for the provision of effective climate services across the Greater Horn of Africa [1].

Long-term sustainability requires embedding climate-health AI competencies into existing academic programs [16]. Proposed interventions include postgraduate certificate programs in “AI for Health Surveillance” targeted at mid-career Ministry of Health epidemiologists, offered by regional universities [19]. The curriculum must cover Python programming for health data, machine learning fundamentals, and climate data processing using satellite earth observations [30]. Furthermore, “Digital Disease Surveillance” should be integrated as an elective track within Master of Public Health programs to ensure alignment with international standards [6, 31]. Undergraduate exposure through “One Health Informatics” modules will create a necessary pipeline for future specialization in environmental health [33].

Bridging the immediate capacity gap while long-term training matures requires structured technical assistance mechanisms [24]. The deployment of “Climate Health Informaticians”—professionals with dual expertise in epidemiology and data science—as two-year embedded advisors within Ministries of Health is essential [24]. These experts should work directly within government organizational charts to co-develop AI models with national staff through hands-on mentorship [1, 10]. Methodological documentation must be transferred in accessible formats, such as Jupyter notebooks, to ensure technical transparency [19]. Regular “code review” sessions will allow local staff to present analytical work for peer feedback, fostering a culture of internal validation [15].

Leveraging existing AI-CHEWS experience elsewhere in Africa can facilitate South-South technical exchanges [5]. This includes three-month fellowships for Somali or South Sudanese health informaticians to work within established regional research teams such as the Kenya Medical Research Institute [25]. Virtual peer mentorship programs can pair district health officers across IGAD countries to share operational lessons on mHealth and alert implementation [14]. Annual regional symposiums on climate-health informatics are necessary to share code, failures, and successes across borders [28]. Formal twinning partnerships between national health institutes will support joint research projects and shared computational infrastructure [4, 37].

To prevent unsustainable licensing costs, technology transfer must prioritize open-source ecosystems [30]. Establishing an IGAD-hosted GitLab instance for all AI-CHEWS code and model documentation will ensure data sovereignty and regional cooperation [29]. Developing a library of pre-trained baseline models allows countries to fine-tune existing algorithms with local data rather than starting from scratch [11]. The distribution of these models as Docker containers facilitates easy deployment across varying technical environments [12]. Additionally, the development of low-code tools can allow epidemiologists to validate basic prediction models without extensive programming knowledge [13].

Technical capacity building fails if trained professionals migrate to the private sector or abroad [26]. Retention requires the creation of specialized “Health Informatician” civil service cadres with competitive compensation benchmarked to the regional tech industry [9]. Clear career progression pathways from district focal points to regional experts must be established based on demonstrated competency [6]. Research incentives, including guaranteed time for publishing operational data, will encourage local analysts to contribute to the global knowledge base [34]. Finally, an IGAD framework allowing for regional mobility will help create a Pan-Horn African professional community [27].

Capacity building monitoring requires indicators beyond simple participation numbers, such as practical assessments of model development skills [32]. Institutional absorption should be measured by the percentage of trained staff remaining in the public health sector over five years [32]. A key sustainability proxy is the number of AI models deployed that were developed primarily by national staff rather than external consultants [19]. Financing this roadmap requires approximately $50–75 million over ten years, necessitating a regional training fund supported by international donors [31]. National governments must also advocate for domestic budget lines that allocate at least 2% of surveillance budgets to digital capacity [8]. Integrating these efforts into existing platforms, such as the Africa CDC, will ensure long-term institutional alignment [37].

### Financing architecture and investment priorities

The development and operationalization of AI-CHEWS across the Horn of Africa requires an estimated $200–300 million over a 10-year horizon, representing a necessary investment to mitigate the escalating climate-driven health crises in the region [31]. While substantial, these costs are modest compared to the economic toll of recurrent epidemics, such as the 2016-17 cholera outbreak in Somalia, which significantly impacted regional health security and productivity [25]. Current financing for health surveillance in the region remains fragmented and project-based, with fewer than 15% of countries meeting international targets for annual surveillance spending [6]. Transitioning to anticipatory, AI-enabled systems requires fundamentally restructured financing mechanisms and a shift away from reactive funding models [1, 11].

A realistic total cost of ownership involves significant initial capital for regional data infrastructure, cloud computing, and interoperability frameworks [30]. The development and validation of disease-specific machine learning models require sustained research and development investment across the IGAD member states [11, 19]. Integration with existing digital health platforms, specifically DHIS2 and national IDSR frameworks, is a primary technical and financial priority [16, 37]. Operational costs, including satellite data subscriptions, embedded technical experts, and continuous model retraining, are estimated to require an additional $15–20 million annually [7, 30]. By coordinating these investments at a regional level, the Horn of Africa can achieve economies of scale and avoid the 40–60% higher costs associated with duplicated country-by-country approaches [1, 29].

Establishing a pooled donor financing mechanism, such as an IGAD Climate-Health Resilience Fund, would harmonize regional efforts and reduce the transaction costs of managing bilateral grants [37]. Such a vehicle provides the 5–7 year funding predictability required for long-term infrastructure and capacity building, which is often impossible under shorter project cycles [1, 31]. These efforts should be linked directly to anticipatory financing instruments, such as parametric insurance, where high-probability AI risk scores trigger automatic payouts for response supplies [11, 32]. Pre-approved contingent credit lines from multilateral banks could also allow governments to draw down emergency funds immediately upon an AI-CHEWS alert, bypassing traditional 6–12 month negotiation delays [7, 29].

National ownership and domestic resource mobilization are essential for the long-term sustainability of the system [9, 28]. IGAD member states should commit to a minimum budget floor for disease surveillance and early warning systems to ensure consistent operational funding [6, 31]. Innovative tax mechanisms, such as levies on the telecom sector, could be dedicated to digital health infrastructure given the reliance of these systems on mobile networks and call detail records [14, 35]. Strategic engagement with the private sector, including agreements with cloud providers for discounted compute credits, can further offset technical costs [30]. Additionally, AI-CHEWS qualifies for climate adaptation finance, and concept notes should be submitted to the Green Climate Fund to frame these systems as critical components of climate-resilient health infrastructure [1, 31].

Investment should be prioritized through a tiered approach, beginning with high-impact, low-cost interventions such as DHIS2 integration and SMS-based alert systems for remote areas [10, 14, 16]. Mid-term priorities include the establishment of sovereign regional data centers to ensure data governance and technical interoperability across borders [29, 37]. Long-term investments should focus on advanced capabilities, including conflict-sensitive models, migration tracking, and the full automation of the satellite-to-supply chain pipeline [7, 25, 30]. Financial sustainability will ultimately rely on documenting the efficiency gains of the system, as every dollar invested in early warning significantly reduces the costs of emergency response and outbreak containment [10, 32]. Framing AI-CHEWS as a regional public good ensures that joint financing remains a collective security priority for all member states [28, 37].

### Multi-stakeholder engagement and political economy

The implementation of AI-CHEWS transcends technical and financial dimensions; its success hinges on navigating complex stakeholder ecosystems and securing sustained political commitment across governments that often face competing crises and limited trust in digital systems [27, 29]. Political will remains the single most critical factor determining whether AI-CHEWS transitions from a localized pilot to sustainable national infrastructure [31]. Establishing joint ministerial steering committees comprising health, environment, and ICT officials is essential to elevate the system to a political priority and ensure cross-sectoral budget allocation [6, 31]. Advocacy at the highest levels must be backed by evidence-based demonstrations, highlighting how predictive systems can reduce outbreak response costs and accelerate case detection compared to traditional surveillance [7, 10, 11]. Parliamentary engagement is further required to secure legislative backing for multi-year budget commitments that survive election cycles and ministerial turnover [29].

Regional political mechanisms provide the necessary platforms for high-level adoption and standard-setting across the Horn of Africa [1]. Leveraging the IGAD Heads of State Summits can facilitate a formal declaration on AI-enabled climate-health resilience, committing member states to minimum standards for data sharing and capacity building [27, 28]. These efforts should be aligned with the African Union’s digital transformation strategy to access continental political momentum and Agenda 2063 health goals [26, 29]. Positioning AI-CHEWS as a flagship example of disease surveillance transformation within the WHO AFRO Regional Committee can generate necessary peer pressure and policy alignment among ministers of health [6, 31].

The emerging technology hubs in Nairobi, Kigali, and Addis Ababa provide a fertile ground for local startups to contribute contextually appropriate innovations that often outperform Global North solutions [26]. Launching a “Climate-Health Tech Challenge” with a dedicated innovation fund can incentivize East African startups to develop low-bandwidth data sync solutions and SMS-based chatbots for community health workers [14, 33]. Selection criteria for such partnerships should prioritize local ownership, open-source commitments, and cost-effectiveness compared to international equivalents [29, 30]. By utilizing regional tech leaders for software engineering talent, AI-CHEWS implementation can employ local developers rather than relying on offshore contractors [15, 26]. Established technology firms should be engaged as partners rather than vendors, securing pro bono cloud compute credits and technical mentorship for government teams [12, 30].

Academic and research institutions are critical for long-term knowledge production and hosting the region’s technical capacity [4, 10]. Designating regional “Climate-Health AI Collaborating Centers” at universities such as Makerere, Addis Ababa, and the University of Nairobi can foster vector ecology and geospatial modeling expertise [4, 22]. These centers require sustained funding to support applied research, graduate student training pipelines, and technical assistance to national governments [19, 37]. Research-to-policy pipelines must be formalized through Science Advisory Boards that present findings directly to health leadership to ensure the rapid adoption of validated innovations [11, 31].

Humanitarian and development partners are both primary funders and end-users of predictive alerts [7]. Involving agencies such as WHO, UNICEF, and MSF in the co-design process ensures that AI-CHEWS generates alerts compatible with existing emergency response protocols and humanitarian cluster coordination [7, 25]. Establishing a donor technical working group is necessary to harmonize investments and avoid duplicative projects with incompatible technical specifications [37]. Operational field testing by NGOs in hard-to-reach areas provides critical feedback on system usability under conflict conditions, ensuring the tools function in settings where they are most needed [25].

Top-down AI systems frequently fail without the buy-in of frontline health workers and local communities [25]. Participatory workshops with district health officers and pastoralist representatives are required to co-define actionable alert thresholds that balance algorithmic sensitivity with operational realism [32]. Integrating two-way communication allows health officers to report alert accuracy, creating a feedback loop that continuously improves model calibration and builds user trust [25, 34]. Furthermore, creating mechanisms to incorporate indigenous early warning knowledge—such as livestock disease patterns—can validate traditional observations rather than replacing them with algorithmic authority [1, 15]. Private sector health actors, including clinic chains and pharmaceutical companies, should also be engaged in data-sharing partnerships to expand surveillance coverage beyond public facilities [37, 39].

Overcoming political barriers, such as mistrust of surveillance and concerns over data sovereignty, requires a transparent and sovereign architecture [29]. All health data should be stored within national or regional data centers with strict residency guarantees to prevent external dependency [27, 28]. Publishing all AI algorithms as open-source code ensures transparency and allows governments to audit model decision-making without external approval [19, 32]. In settings of political tension, positioning AI-CHEWS as neutral technical infrastructure operated by regional bodies like Africa CDC can mitigate fears of political monitoring [29]. Finally, long-term political sustainability relies on building domestic constituencies—including science journalists and civil society—who can advocate for continued government commitment and budget allocation [6, 31].

## Policy roadmap

We propose a phased implementation roadmap anchored in the IGAD framework for the transition from fragmented pilots to a cohesive regional system. The phased implementation plans and associated stakeholders are summarized in Table 2.

**Table 2:** Implementation roadmap

Phase	Key Actions	Capacity Building & Tech Transfer	Financing & Governance	Lead Stakeholders
Short-term (0–2 years)	• Data Harmonization Pilots: Standardize historical climate-health datasets in 3–5 pilot cross-border districts • Low-Tech Alerts: Operationalize SMS alerts based on simple rainfall thresholds (NDVI) • DHIS2-CHEWS Integration: Deploy CHEWS module in pilot districts • AI Training Infrastructure: Establish regional GitLab repository, begin model development	• Launch “Climate Health Data Fellowships” for 50 MOH staff • Embedded Experts Program: Deploy 10–15 technical advisors in ministries • University Curriculum Integration: Introduce modules in 3 regional universities • South-South Exchanges: Initiate Kenya-Somalia-Ethiopia technical visits	• Establish IGAD Climate-Health Trust Fund (target: $40 M capitalization) • Joint Ministerial Steering Committees in all countries • Draft IGAD Data Sharing Treaty • Secure initial climate finance ($10 M Green Climate Fund)	Ministries of Health, WHO, ICPAC, Africa CDC, National Universities, Bilateral Donors
Mid-term (3–5 years)	• National CHEWS Rollout: Deploy validated AI models to national data centers in all IGAD states • EO Integration: Automate satellite data ingestion into DHIS2 • Anticipatory Financing: Link alerts to pre-arranged disaster financing triggers • Local Startup Engagement: Launch Climate-Health Tech Challenge with $5 M innovation fund • Conflict-Sensitive Pilots: Deploy in South Sudan, Somalia with federated architecture	• Postgraduate Certificate Programs operational at 3 universities (Nairobi, Addis, Khartoum) • Establish 3 regional Centers of Excellence • Twinning Partnerships with KEMRI, NICD South Africa • Train-the-trainer program reaching 500 district health officers • Regional CHEWS Symposium (annual)	• IGAD Data Sharing Treaty ratified and legally binding • National budget allocations ≥ 5% of health budgets to surveillance • Parametric insurance products launched (African Risk Capacity) • Private sector partnerships (cloud credits, telecom data sharing) • Regional data center operational (IGAD-hosted)	Universities, Donors (World Bank, Gavi), National Met Agencies, Private Sector (Microsoft, Safaricom), Insurance Sector, African Risk Capacity
Long-term (5–10 years)	• Regional AI Network: Establish trans-boundary, autonomous forecasting hub at IGAD • Full Automation: End-to-end integration from satellite signal to supply chain logistics • Multi-Disease Integration: Expand beyond initial focus diseases to NCDs, AMR surveillance • Advanced Capabilities: Conflict-migration models, sub-district hotspot forecasting	• 200 + nationals with AI-health competencies employed in public sector • MPH specialization tracks in 5 + universities • Low-code CHEWS tools allowing non-programmers to develop models • Regional professional association (Horn Africa Climate-Health Informaticians)	• Governance: Legally binding regional data-sharing treaty enforced • Sustainable Financing: 60% domestic, 40% external • Cost-recovery mechanisms for commercial users • Full regulatory frameworks (AI certification, liability) • Evidence: ≥5 peer-reviewed impact evaluations demonstrating cost-effectiveness	IGAD, Africa CDC, African Union, Private Sector Tech Partners, Regional Universities, National Governments (domestic financing)

### Strategic enabler: decolonizing infrastructure

To ensure sustainability, the roadmap prioritizes the development of sovereign computational infrastructure. This involves shifting away from exclusive reliance on foreign cloud providers toward establishing regional data centers and open-source algorithmic libraries managed by IGAD member states. The ‘sovereign AI’ approach is critical to insulating the region’s early warning capabilities from global geopolitical fluctuations and ensuring that access to life-saving intelligence is not determined by external market forces.

## Research agenda

Operationalizing this roadmap requires a targeted research agenda to close the evidence gap. First, we need rigorous Comparative AI Performance studies to benchmark which algorithms (e.g., Random Forest vs. LSTM) perform best, specifically in the arid and semi-arid climates of Horn, rather than assuming that models from tropical Asia will transfer directly [17]. Second, research must focus on Conflict & Migration Integration and develop proxies for health system functionality in war zones to correct model bias.

GeoAI Flood–Disease Interaction Models require specific attention to understand the lag time between extreme flooding and outbreaks of vector-borne versus water-borne diseases in this specific topography [4]. Crucially, we need CHEWS Health-Impact Trials—cluster-randomized controlled trials that do not just measure model accuracy (AUC) but also measure the clinical outcome: did the early warning actually result in reduced morbidity? Third, research into sustainable capacity-building models is needed to identify the most effective ways to retain local data science talent within the public sector.

Fourth, AI Implementation Science Studies are needed to rigorously evaluate real-world deployment: What are the barriers to district health officers acting on AI-generated alerts? How do alert false-positive rates affect user trust and alert fatigue over time? What institutional factors predict successful DHIS2-CHEWS integration versus failed adoption? These qualitative and mixed-methods studies are as critical as algorithm optimization.

Fifth, Conflict-Sensitive Model Development Research must address bias in non-random missing data. When conflict zones lack surveillance, standard imputation techniques fail. Research into Bayesian hierarchical models that explicitly model the missingness mechanism, or transfer learning approaches that train on stable regions then adapt to conflict zones using sparse data, could improve equity in outbreak prediction for the most vulnerable populations.

Sixth, Long-Term Sustainability and Cost-Effectiveness Research: Prospective economic evaluations comparing total costs (capital + operational) and outcomes (outbreaks averted, DALYs saved, response cost reductions) between AI-CHEWS versus conventional surveillance. This evidence is essential for securing sustained domestic financing and demonstrating value to finance ministries skeptical of technology investments.

Finally, Ethical AI Governance Research: Empirical studies on data sovereignty preferences among affected populations (do refugees consent to CDR-based mobility tracking if it improves outbreak response? ), optimal governance structures that balance privacy with public health, and evaluation of participatory model development approaches versus technocratic design.

## Conclusion

Technology for predicting climate-sensitive outbreaks in the Horn of Africa is currently available. AI offers a powerful lens to view the “fog of war” and climate chaos, transforming noisy environmental signals into lifesaving foresight. However, for this promise to be realized, the approach must be regionally owned, ethically governed, and integrated into the existing health architecture, and not superimposed upon it. The cost of inaction is measured in the lives lost due to preventable epidemics. IGAD and African scientific leaders have the opportunity to pioneer a model of “digital humanitarianism” that is robust enough for future climate shocks.

Operationalizing this vision requires simultaneous progress across technical, institutional, financial, and political dimensions. The technical building blocks—machine learning algorithms, satellite earth observation, mobile connectivity—exist and have demonstrated efficacy in other regions. What the Horn of Africa lacks is not technological capability but rather coordinated investment in regional digital infrastructure, systematic capacity building that creates sustainable local expertise, financing mechanisms that transcend short-term project cycles, and political commitment to data sharing across sovereign boundaries. The roadmap presented here is ambitious but achievable: phased implementation beginning with low-cost, high-impact interventions (SMS alerts, DHIS2 integration), building evidence and constituencies for sustained investment, and ultimately establishing autonomous regional forecasting capacity. Success requires embracing the paradox of digital humanitarianism in fragile states—systems must be sophisticated enough to navigate conflict and data scarcity, yet simple enough to function when Internet fails and governments collapse. By prioritizing sovereignty (local data stays local), transparency (open-source algorithms), equity (conflict-zone coverage), and participation (communities co-design thresholds), AI-CHEWS can avoid reproducing the extractive patterns of past technological interventions. The time required to build a digital shield is now.

## Data Availability

No datasets were generated or analyzed for this study.
